# Gene duplications resulting in over expression of glucokinase are not a common cause of hypoglycaemia of infancy in humans

**DOI:** 10.1016/j.ymgme.2008.01.008

**Published:** 2008-06

**Authors:** Martijn van de Bunt, Emma L. Edghill, Khalid Hussain, Sian Ellard, Anna L. Gloyn

**Affiliations:** Diabetes Research Laboratories, Oxford Centre for Diabetes, Endocrinology & Metabolism, Churchill Hospital Old Road, Headington, Oxford, UK; Institute of Biomedical & Clinical Science, Peninsula Medical School, Exeter, UK; Institute of Child Health, Great Ormond Street Hospital, London, UK; Institute of Biomedical & Clinical Science, Peninsula Medical School, Exeter, UK; Diabetes Research Laboratories, Oxford Centre for Diabetes, Endocrinology & Metabolism, Churchill Hospital Old Road, Headington, Oxford, UK

It is well established that chromosome rearrangements and duplications which result in the loss of or the over expression of gene(s) can result in syndromes which feature hypoglycaemia [1,2]. More recently studies have demonstrated the importance of “gene dosage” in monogenic disorders of the pancreatic β-cell [3,4]. Investigators routinely screen for mutations using the gold standard methodology of direct sequencing of the coding regions and conserved splice sites of the gene of interest, however this method does not detect heterozygous deletions or duplications of one or more exons of the gene. *Hypoglycaemia of infancy (HI)* is a rare condition characterised by unregulated insulin secretion despite hypoglycaemia. Mutations in genes encoding the K_ATP_-channel subunits SUR1(*ABCC8*) and Kir6.2 (*KCNJ11*), glucokinase (*GCK*), the mitochondrial enzymes glutamate-dehydrogenase (*GLUD1*) and short-chain 3-hydroxyacyl-CoA-dehydrogenase (*SCHAD*) are responsible for approximately 50% of the cases of *HI* [5]. *GCK* activating mutations have been reported in a relatively small number of cases (*n* = 7) of *HI* and are characterised by regulated insulin secretion albeit at a reduced threshold for glucose stimulated insulin release [6]. The majority of patients can be treated successfully with the potassium channel opener diazoxide or with diet treatment alone, although cases that have undergone partial pancreatectomy have been reported [7].

A number of studies in rodents have shown than over expression of the glucokinase gene results in increased enzyme activity [8–10]. Over expression resulting from *GCK* gene duplications would result in increased liver and β-cell glucokinase activity and could therefore result in hypoglycaemia. We hypothesised that duplications of *GCK* might play a role in the pathogenesis of *HI* and account for the significant number of cases of *HI* of unknown genetic aetiology. To determine the putative role of partial or whole gene duplications of *GCK*, we screened the *GCK* gene for copy number variation using a multiplex ligation-dependent probe amplification (MLPA) assay in unrelated subjects with unexplained *HI* (*n* = 33). The clinical characteristics of the patients screened are shown in Table 1. The MLPA assays were performed using a synthetic *GCK* probe mix as reported by Ellard et al. and the data was analysed as described by Lai et al. [4,11]. No duplications of the *GCK* gene were detected in the 33 patients screened. Our data therefore suggest that over expression of glucokinase is unlikely to be a common cause of *HI* in humans but does not exclude rare cases where *GCK* duplications may contribute to a lowering of blood glucose levels. In conclusion, there is no evidence to support including a *GCK* dosage assay in the diagnostic testing repertoire for *HI*.

## Figures and Tables

**Table 1:** Clinical Characteristics of subjects studied

Variable	
*n*	33
% Male	48.5
Age at diagnosis (days)	139.1 (336.5)
∗Age at examination (yrs)	6.1 (5.8)
Fasting glucose (mmol/L)	2.02 (0.57)
Current treatment (%) (diazoxide/pancreatectomy/octreotide/diet/not known)	45/36/6/3/9
Birth weight (g)	3361.7 (823.3)
% With birth weight over 70th percentile	51

Data are mean (SEM).

∗ Fasting glucose measurement.
